# MCF2L-AS1 promotes the biological behaviors of hepatocellular carcinoma cells by regulating the miR-33a-5p/FGF2 axis

**DOI:** 10.18632/aging.204795

**Published:** 2023-07-10

**Authors:** Hongliang Ou, Yunsong Qian, Li Ma

**Affiliations:** 1 Department of Liver Diseases, Ningbo No.2 Hospital, University of Chinse Academy of Sciences, Ningbo 315000, Zhejiang, P.R. China

**Keywords:** hepatocellular carcinoma, MCF2L-AS1, miR-33a-5p, FGF2

## Abstract

Long noncoding RNA MCF2L-AS1 functions in the development of cancers like lung cancer, ovarian cancer, and colorectal cancer. Notwithstanding, its function in hepatocellular carcinoma (HCC) stays obscure. Our research probes its role in MHCC97H and HCCLM3 cell proliferation, migration, and invasion. qRT-PCR gauged MCF2L-AS1 and miR-33a-5p expressions in HCC tissues. CCK8, colony formation, Transwell, and EdU assays detected HCC cell proliferation, invasion, and migration, respectively. The xenograft tumor model was built to confirm the MCF2L-AS1-mediated role in HCC cell growth. Western blot and immunohistochemistry detected FGF2 expression in HCC tissues. Bioinformatics analysis predicted the targeted relationships between MCF2L-AS1 or FGF2 and miR-33a-5p, which were further examined through dual-luciferase reporter gene and pull-down assays. MCF2L-AS1 was expressed highly in HCC tissues and cells. MCF2L-AS1 upregulation enhanced HCC cells’ proliferation, growth, migration, and invasion and reduced apoptosis. miR-33a-5p was demonstrated as an underlying target of MCF2L-AS1. miR-33a-5p impeded HCC cells’ malignant behaviors. MCF2L-AS1 overexpression reversed miR-33a-5p-mediated effects. MCF2L-AS1 knockdown enhanced miR-33a-5p and negatively regulated FGF2 protein. miR-33a-5p targeted and inhibited FGF2. miR-33a-5p overexpression or FGF2 knockdown inhibited MCF2L-AS1-mediated oncologic effects in MHCC97H. By modulating miR-33a-5p/FGF2, MCF2L-AS1 exerts a tumor-promotive function in HCC. The MCF2L-AS1-miR-33a-5p-FGF2 axis may provide new therapeutic targets for HCC treatment.

## INTRODUCTION

Hepatocellular carcinoma (HCC) is regarded as one of the most prevalent cancers across the world with high morbidity, aggressive invasion, rapid development, and poor prognosis [1, 2]. Due to the high incidence of hepatitis B virus (HBV) infection, China has a high prevalence of hepatocellular carcinoma (HCC) [3]. Patients with HCC are prone to show malignant clinicopathological features in lymphatic vessels, blood vessels and distant viscera metastasis at the early stage [4]. Elucidating the molecular biological changes in HCC development can help HCC diagnosis and treatment [5, 6].

Fibroblast growth factors (FGFs) are found to be extensively expressed in the tissues of human body; FGF and FGF receptor (FGFR) usually constitute FGFR signaling pathways to participate in growth and development, wound healing and fibrosis, inflammation, and neogenesis of many tumors [7, 8]. FGF2, belonging to the FGF family, is situated on human chromosome 4q26 with a total length of 38kb [9]. FGF2 is originally isolated and purified from the pituitary gland of cattle and undertakes a significant role in promoting cellular proliferation, differentiation, and vascular growth [10]. Growing evidence has shown that FGF2 is altered in tumor tissues [11]. For instance, FGF2 expression was increased in ovarian cancer (OC), which was related to the clinical stage [12, 13]. FGF2 could interact with FGFR to trigger downstream signaling and regulate HCC invasiveness [14, 15].

miRNAs are thought to impact over 90% of protein-coding gene loci in the human genome through diverse regulatory approaches, such as combining with the 3’UTR of target messenger RNAs (mRNAs) and affecting gene expression post-transcriptionally [16, 17]. miR-33a-5p, one member of miRNAs, participates in tumor progression. For example, miR-33a-5p targets Snail family transcriptional repressor 2 (SNAI2) to suppress the migration of melanoma cells [18]. Xing et al. have discovered that miR-33a-5p can mediate the proliferation, migration and apoptosis of cardiomyocyte progenitor cells via targeting NODAL modulator 1 (NOMO1) [19]. On the other hand, long non-coding RNAs (lncRNAs), another class of ncRNAs with over 200 nts in length with tissue specificity [20], undertake an essential role in many biological behaviors including tumorigenesis, neuroscience and ontogeny, especially in tumor cell proliferation, apoptosis, invasion, migration and chemotherapeutic drug resistance [21–24]. lncRNA MCF2L antisense RNA 1 (MCF2L-AS1) is a newly reported molecule in recent years. Kong et al. indicated that MCF2L-AS1 aggravates colorectal cancer development via miR-105-5p/RAB22A signaling [25]. lncRNA MCF2L-AS1 exhibits a high level under the conditions of non-small cell lung cancer, and knocking down MCF2L-AS1 impedes the cancer stem cell (CSC)-like traits of NSCLC cells [26]. However, lncRNA MCF2L-AS1 profile in both liver cancer tissues and cells, as well as its regulatory mechanism, is still poorly understood.

The intention of the research is to delve into the influence of MCF2L-AS1, miR-33a-5p and FGF2 on liver cancer cell proliferation, invasion, migration and apoptosis, as well as to examine the regulatory mechanism of the MCF2L-AS1-miR-33a-5p-FGF2 axis in the context of HCC. We hope this study provides a new strategy for HCC treatment.

## RESULTS

### lncRNA MCF2L-AS1 was altered in hepatocellular carcinoma

**Figure 1:**
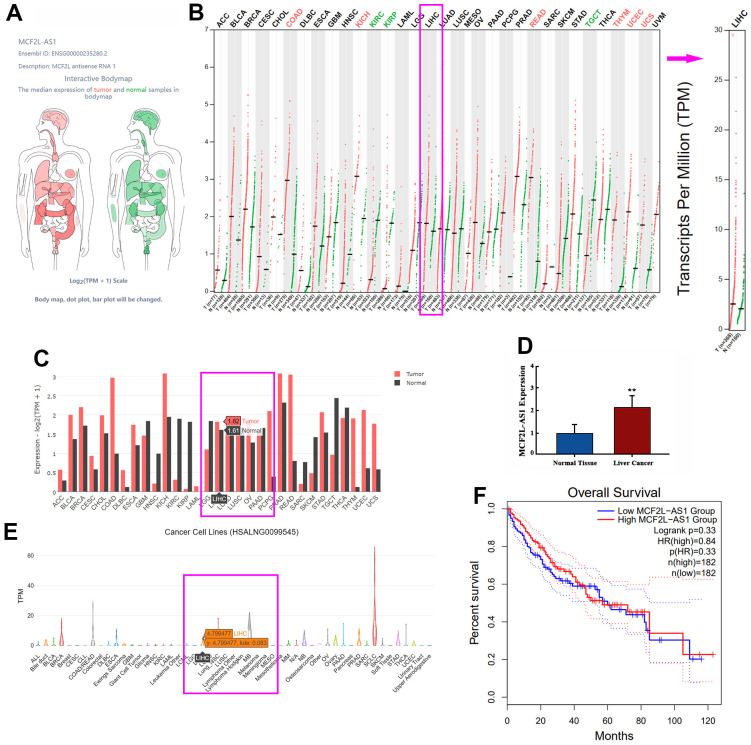
**The profile of lncRNA MCF2L-AS1 in hepatocellular carcinoma.** (**A**) Schematic diagram showed MCF2L-AS1 expression in various organs in the human body. (**B**, **C**) The database showed MCF2L-AS1 expression in normal tissues and liver cancer tissues. (**D**) qRT-PCR detected MCF2L-AS1 expression levels in HCC tissues and adjacent normal tissues. (**E**) The database showed MCF2L-AS1 expression in liver cancer cells. (**F**) K-M plotter showed the correlation between the MCF2L-AS1 level and the survival rate of HCC patients. (**, P<0.01, the difference is statistically significant). All experiments were duplicated 3 times.

The GEPIA database confirmed that lncRNA MCF2L-AS1 has a certain expression ability in most organs in the human body, including the liver (Figure 1A). Enhanced MCF2L-AS1 expression in liver cancer tissue samples (Figure 1B, 1C). We also detected MCF2L-AS1 expression in tissue samples from liver cancer patients by qRT-PCR, unveiling that MCF2L-AS1 profile was vigorously up-regulated vis-à-vis the normal control group (Figure 1D). Through the database, we also found MCF2L-AS1 could also be expressed in liver cancer cells (Figure 1E). In addition, an elevated MCF2L-AS1 profile bore a relation to the poorer survival rate of liver cancer patients (Figure 1F). Therefore, lncRNA MCF2L-AS1 might act as a tumor-promoting factor in liver cancer.

### lncRNA MCF2L-AS1 exerted tumor-promotive effects in hepatocellular carcinoma cells *in vitro*

**Figure 2:**
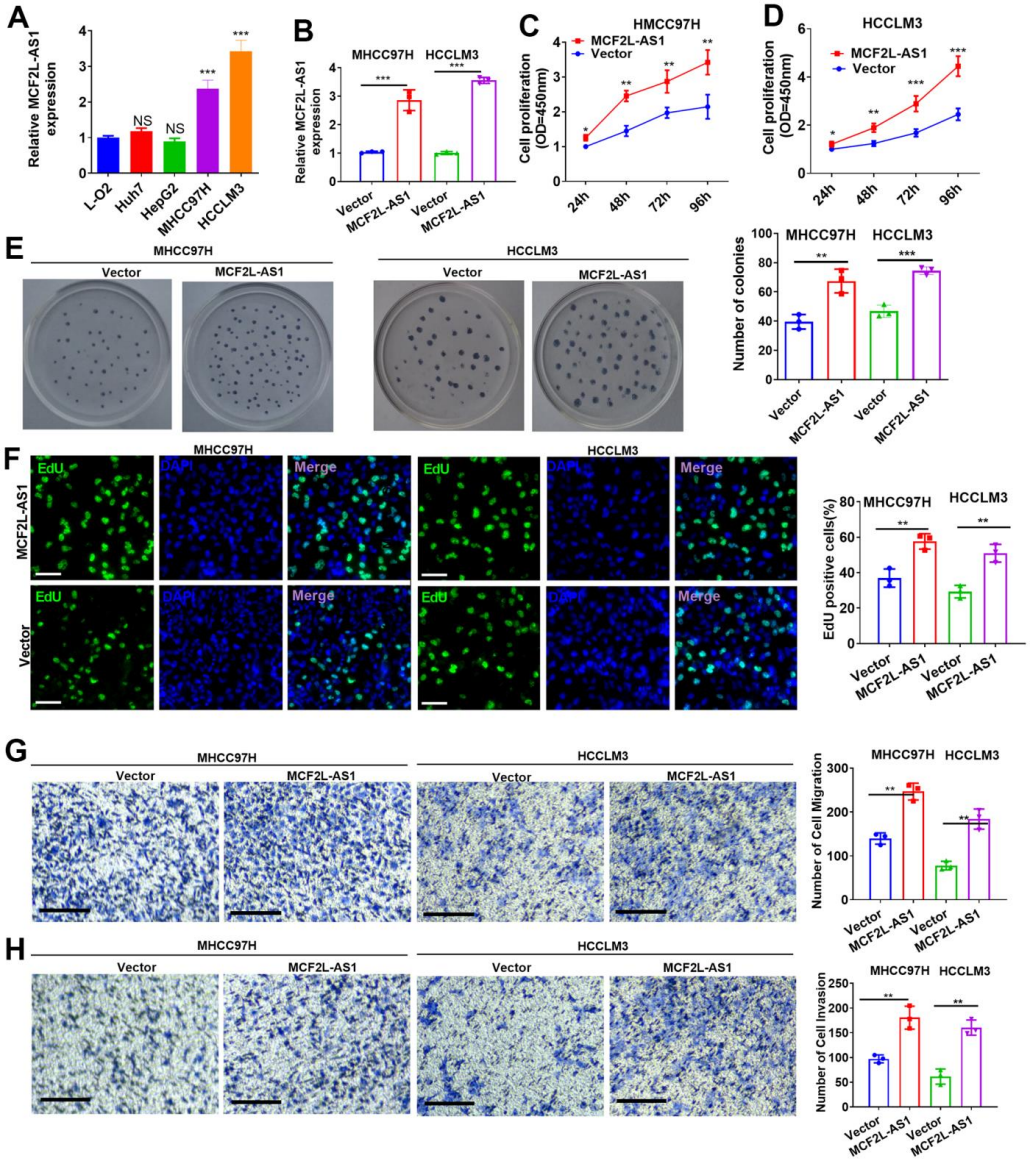
**Effects of MCF2L-AS1 on cell proliferation, invasion and apoptosis.** MCF2L-AS1 overexpression plasmids were transfected into MHCC97H and HCCLM3 cells. (**A**, **B**) MCF2L-AS1 profile was detected through qRT-PCR. (**C**, **D**) CCK8 assay detected MHCC97H and HCCLM3 cell proliferation. (**E**) Colony formation assay was conducted for detecting cell colony formation ability. (**F**) EdU staining was performed for evaluating cell proliferation. Scale bar=50 μm. (**G**, **H**) MHCC97H and HCCLM3 cell migration and invasion were tracked by Transwell. Scale bar=200 μm. * P<0.05, **P<0.01, *** P<0.001. N=3.

We have detected MCF2L-AS1 in four HCC cell lines, including MHCC97H, HCCLM3, Huh7 and HepG2, and human Normal Liver Cells L-O2. MCF2L-AS1 has significant upregulation in MHCC97H and HCCLM3 cells (Figure 2A). The MCF2L-AS1 overexpression model was constructed in both MHCC97H and HCCLM3 cells (Figure 2B). CCK8 assay, colony formation, and EdU staining assay examined cell proliferation. Those assays all unraveled that overexpressing MCF2L-AS1 bolstered cell proliferative abilities (versus the Vector group) (Figure 2C–2F). Next, the migrative and invasive abilities were assessed. As shown by the data, MCF2L-AS1 overexpression led to enhanced migration and invasion in MHCC97H and HCCLM3 cells (Figure 2G, 2H).

### lncRNA MCF2L-AS1 promoted tumor cell growth *in vivo*

**Figure 3:**
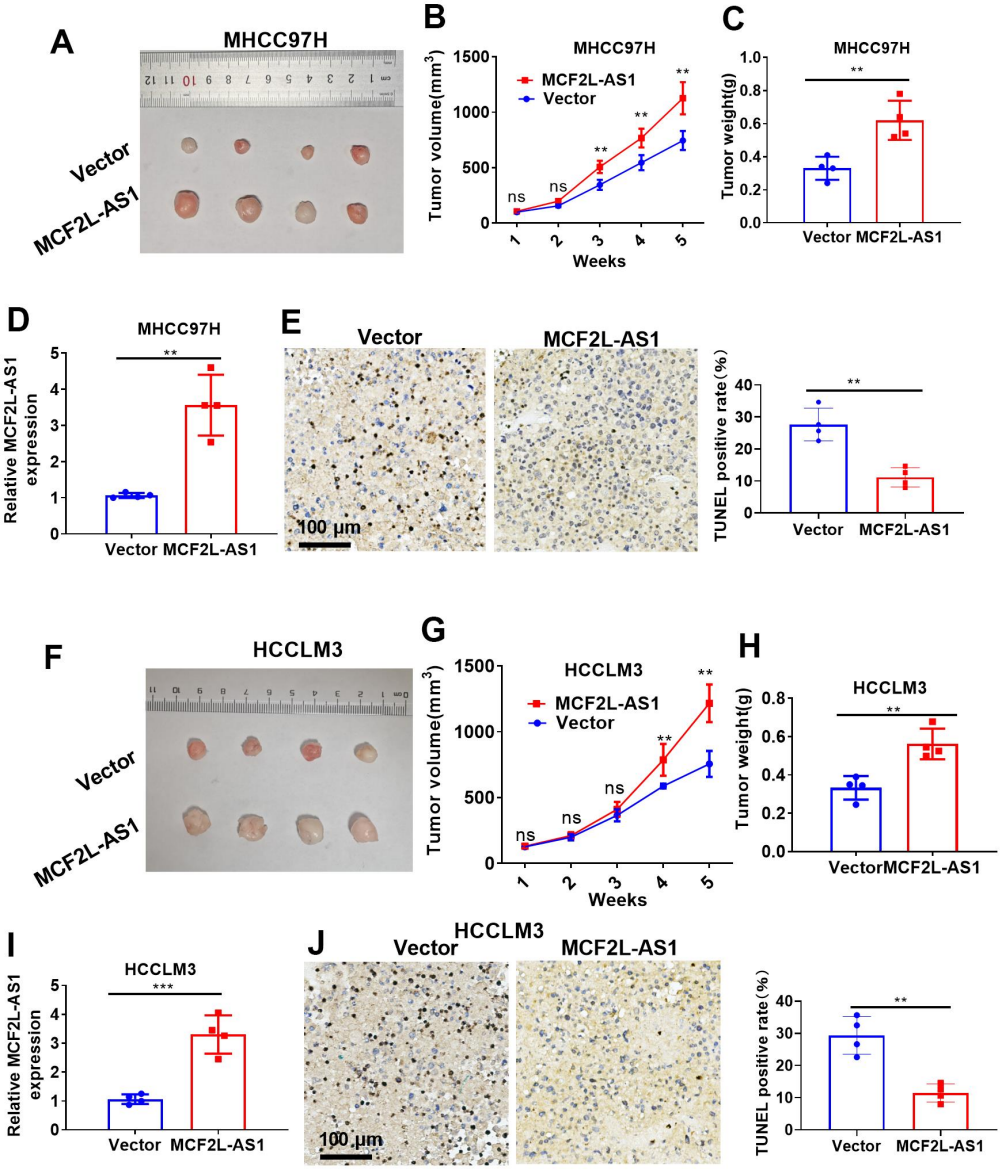
**The effects of MCF2L-AS1 on HCC cell growth *in vivo*.** MHCC97H and HCCLM3 cells were transfected along with MCF2L-AS1 overexpression plasmids and used for *in vivo* experiments. (**A**) Tumor images. (**B**) Tumor volume. (**C**) Weight of tumor. (**D**) RT-PCR was conducted for detecting MCF2L-AS1. (**E**) TUNEL staining kit was used for detecting cell apoptosis. (**F**) Tumor images. (**G**) Tumor volume. (**H**) Tumor weight. (**I**) RT-PCR was conducted for detecting MCF2L-AS1. (**J**) The TUNEL staining kit was used for detecting cell apoptosis. Scale bar=100 μm. Ns P>0.05, **P<0.01, ***P<0.001. N=4.

Next, we conducted *in vivo* assays for investigating the function of MCF2L-AS1. As suggested by our statistics, MCF2L-AS1 overexpression enhanced tumor volume and weight in the xenograft tumor model (Figure 3A–3C). RT-PCR confirmed that MCF2L-AS1 was upregulated in the tumor tissues (versus the Vector group, Figure 3D). Next, the TUNEL assay was performed for evaluating apoptosis. It was found that MCF2L-AS1 overexpression reduced TUNEL-positive cell rate (Figure 3E). Furthermore, we performed *in vivo* assays using HCCLM3 cells. Similarly, MCF2L-AS1 overexpression promoted cell growth and reduced apoptosis (Figure 3F–3J). Therefore, lncRNA MCF2L-AS1 showed tumor-promotive effects in liver cancer.

### miR-33a-5p was a target of lncRNA MCF2L-AS1

**Figure 4:**
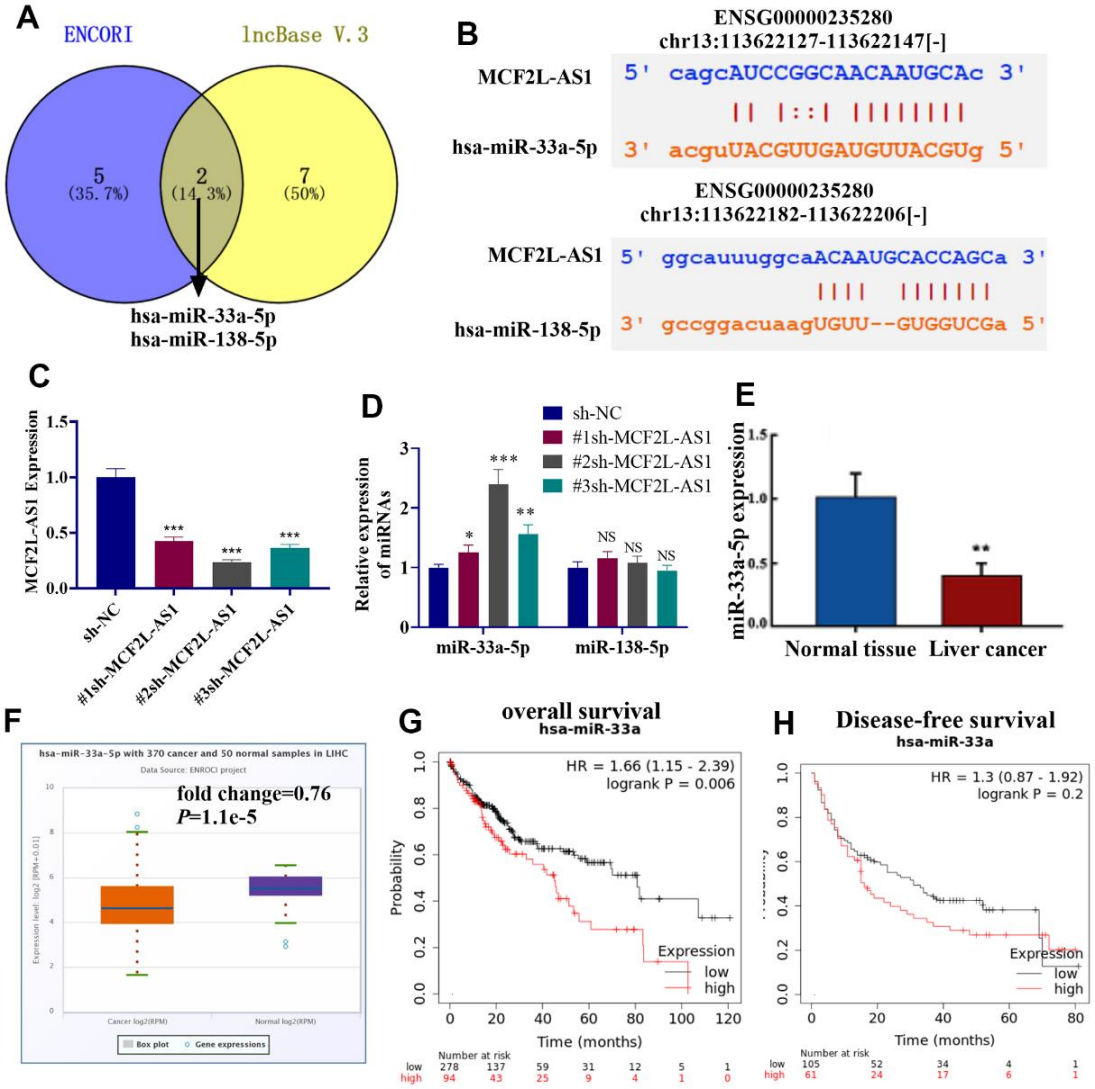
**miR-33a-5p was a potential target of MCF2L-AS1.** (**A**) The potential miRNA targets of lncRNA MCF2L-AS1 were predicted through two online databases, including ENCORI (https://starbase.sysu.edu.cn/) and lncBase V.3 (https://diana.e-ce.uth.gr/lncbasev3). The common miRNAs were analyzed via Venny’s diagram. (**B**) The binding sites of the two miRNAs on MCF2L-AS1 were shown. (**C**) The MCF2L-AS1 downregulation model was constructed. (**D**, **E**) MCF2L-AS1, miR-33a-5p and miR-138-5p levels were gauged via qRT-PCR. (**F**) The miR-33a-5p level in liver hepatocellular carcinoma (LIHC) tissues from the TCGA database were analyzed via ENCORI (https://starbase.sysu.edu.cn/). (**G**, **H**) K-M plotter evaluated the relationship between the miR-33a-5p level and the overall survival of LIHC patients.

Though two online databases, including ENCORI (https://starbase.sysu.edu.cn/) and lncBase V.3 (https://diana.e-ce.uth.gr/lncbasev3), potential miRNA targets of lncRNA MCF2L-AS1 were predicted. 8 miRNAs were found in the ENCORI database, and 9 miRNAs were found in lncBase V.3. Those miRNAs were listed in Table 1. Venny’s diagram showed that two miRNAs (miR-33a-5p and miR-138-5p) were common in the two databases (Figure 4A). The binding sites of the two miRNAs on MCF2L-AS1 were shown (Figure 4B). The MCF2L-AS1 downregulation model was built and validated via qRT-PCR (Figure 4C). By examining miR-33a-5p and miR-138-5p in HCC cells with MCF2L-AS1 knockdown, we uncovered that miR-33a-5p was substantially upregulated (miR-138-5p’s level was not significantly altered) (Figure 4D). qRT-PCR exhibited a decrease in miR-33a-5p’s profile in liver cancer tissues vis-à-vis normal tissues (Figure 4E). miR-33a-5p’s level in liver hepatocellular carcinoma (LIHC) tissues from the TCGA database were analyzed via ENCORI (https://starbase.sysu.edu.cn/). The result displayed that versus normal tissues, miR-33a-5p was dramatically downregulated in LIHC tissues (P=1.1e5, Figure 4F). Moreover, higher miR-33a-5p predicted the poorer overall survival of LIHC patients (Figure 4G, 4H). Collectively, miR-33a-5p was a potential miRNA target of MCF2L-AS1 and got involved in HCC progression.

**Table 1:** Potential miRNA targets of MCF2L-AS1.

**ENCORI**	**lncBase V.3**
hsa-miR-33a-5p	hsa-let-7a-5p
hsa-miR-874-3p	hsa-let-7d-5p
hsa-miR-514a-5p	hsa-let-7e-5p
hsa-miR-138-5p	hsa-let-7f-5p
hsa-miR-33b-5p	hsa-miR-1-3p
hsa-miR-33a-5p	hsa-miR-138-5p
hsa-miR-7853-5p	hsa-miR-210-3p
hsa-miR-105-5p	hsa-miR-33a-5p
	hsa-miR-98-5p

### lncRNA MCF2L-AS1 reversed the antitumor effects of miR-33a-5p in hepatocellular carcinoma cells

**Figure 5:**
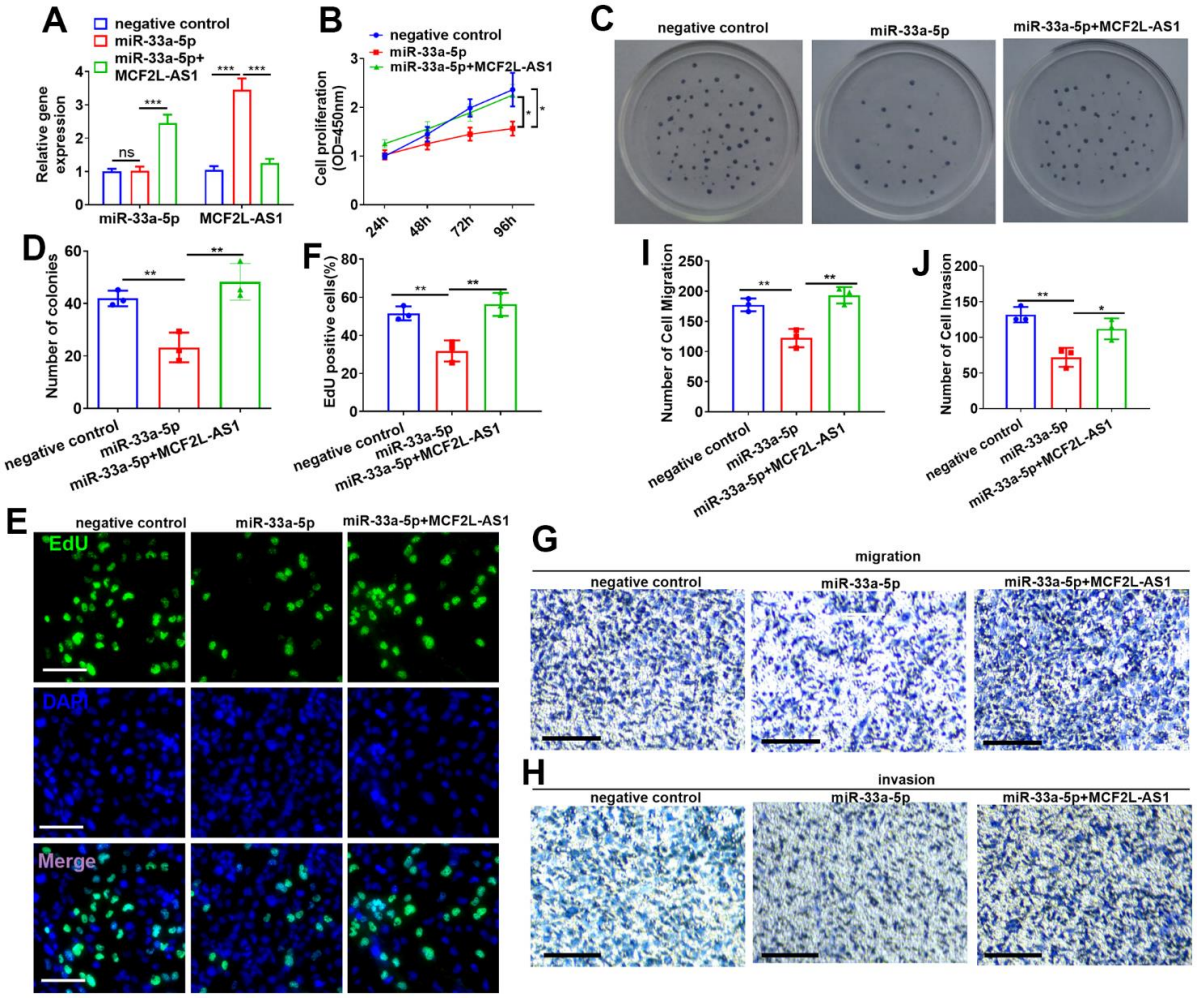
**Effects of MCF2L-AS1-miR-33a-5p on MHCC97H cell proliferation, invasion and apoptosis.** miR-33a-5p mimics and/or MCF2L-AS1 overexpression plasmids were transfected into MHCC97H cells. (**A**) miR-33a-5p and MCF2L-AS1 expression levels were verified by qRT-PCR. (**B**) CCK8 detected MHCC97H and HCCLM3 cell proliferation. (**C**, **D**) Colony formation experiment was conducted for detecting cell colony formation ability. (**E**, **F**) EdU staining was performed for evaluating cell proliferation. Scale bar=50 μm. (**G**–**J**) Transwell assay detected MHCC97H cell migration and invasion, Scale bar=200 μm. * P<0.05, **P<0.01, *** P<0.001. N=3.

The overexpressed model of miR-33a-5p was constructed in MHCC97H cells, and MCF2L-AS1 overexpression plasmids were co-transfected. MCF2L-AS1 and miR-33a-5p levels were detected. As indicated by the data, miR-33a-5p mimics didn’t alter MCF2L-AS1’s level. However, MCF2L-AS1 overexpression led to reduced miR-33a-5p (vs. the miR-33a-5p group, Figure 5A). CCK8, colony formation, and EdU staining assays were capitalized for the examination of cell proliferation. These outcomes verified that overexpressing miR-33a-5p attenuated the proliferative levels of MHCC97H cells. MCF2L-AS1 overexpression promoted cell proliferative abilities (compared with the miR-33a-5p group) (Figure 5B–5F). The migrative and invasive abilities were detected via Transwell. Consequently, miR-33a-5p dampened migration and invasion in MHCC97H cells, and this effect was reversed by MCF2L-AS1 (Figure 5G–5J).

### lncRNA MCF2L-AS1-miR-33a-5p-FGF2 formed a regulatory axis in HCC

**Figure 6:**
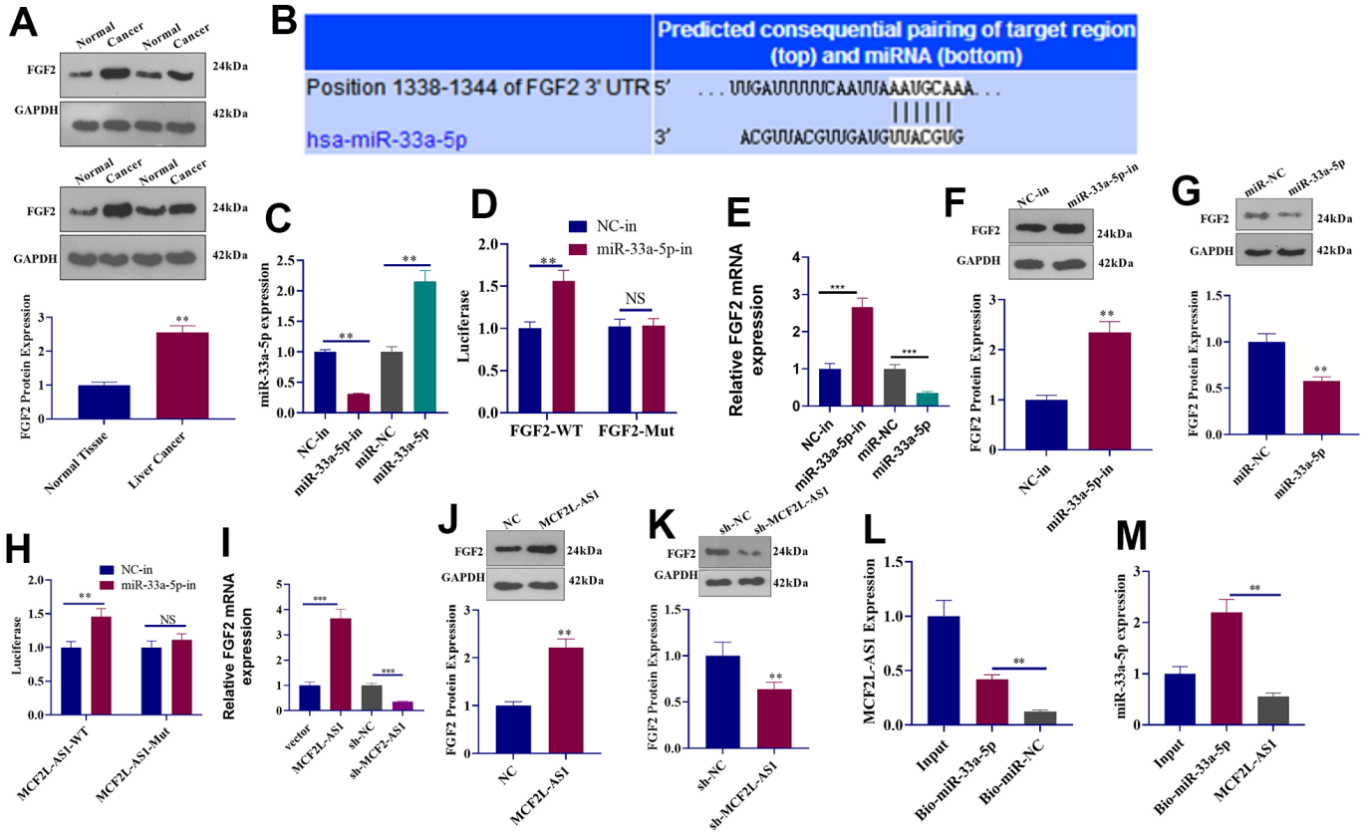
**The regulatory mechanism of the lncRNA MCF2L-AS1-miR-33a-5p-FGF2 axis.** (**A**) Western blot detected FGF2 protein expression in liver cancer tissues. (**B**) Targetscan forecast a binding site between miR-33a-5p and FGF2. (**C**) qRT-PCR checked the transfection efficiency of miR-33a-5p inhibitors and mimics. (**D**) The luciferase activity of 293T cells transfected with different vectors was confirmed through dual-luciferase reporter assay. (**E**) qRT-PCR checked the mRNA level of FGF2. (**F**, **G**) Western blot detected FGF2 protein expression. (**H**) The luciferase activity of 293T cells transfected with MCF2L-AS1-WT, MCF2L-AS1-Mut, the miR-33a-5p inhibitor or negative control was ascertained through dual-luciferase reporter assay. (**I**) qRT-PCR checked the mRNA level of FGF2. (**J**, **K**) Western blot detected FGF2 protein expression. (**L**, **M**) Pull-down assay and qRT-PCR detected the regulatory relationship between MCF2L-AS1 and miR-33a-5p. (**, P<0.01, the difference is statistically significant).

Western blot showed that as opposed to adjacent normal tissues, an increased expression of FGF2 protein was detected in liver cancer tissues (Figure 6A). Through the Targetscan database, we uncovered that miR-33a-5p had binding sites on FGF2 mRNA (Figure 6B). To verify their targeting relationship, we transfected miR-33a-5p-in (inhibitor) or mimics into MHCC97H cells. The experiment of qRT-PCR indicated miR-33a-5p profile was vigorously decreased by miR-33a-5p-in and promoted by miR-33a-5p mimics (Figure 6C). Dual-luciferase reporter assay indicated that miR-33a-5p-in could increase the luciferase activity of FGF2-WT plasmids-transfected 293T cells, but it did not change the luciferase activity of FGF2-Mut plasmids-transfected 293T cells (Figure 6D). qRT-PCR and Western blot denoted that miR-33a-5p downregulation suppressed the level of FGF2 mRNA and protein (Figure 6E–6G), while miR-33a-5p promotion reduced FGF2 mRNA and protein expression (Figure 6E–6G). After carrying out dual-luciferase reporter assay and Western blot, we uncovered that miR-33a-5p-in elevated the luciferase activity of MCF2L-AS1-WT plasmids-transfected 293T cells, whereas it failed to alter the luciferase activity of MCF2L-AS1-Mut plasmids-transfected 293T cells (Figure 6H). Overexpression of MCF2L-AS1 positively regulated the FGF2 mRNA and protein level (Figure 6I–6K), while knockdown MCF2L-AS1 could negatively regulate FGF2 expression (Figure 6I–6K). Pull-down assay indicated miR-33a-5p lowered MCF2L-AS1 expression in MHCC97H cell lysate (Figure 6L), and transfection of MCF2L-AS1 overexpression plasmids in MHCC97H cells could counteract the elevated level of miR-33a-5p induced by mimic (Figure 6M). These results suggested that MCF2L-AS1 competitively combined with miR-33a-5p and up-regulated FGF2 to form a complex regulatory axis.

### Effects of MCF2L-AS1-miR-33a-5p-FGF2 axis on MHCC97H cellular proliferation, migration, and invasion

**Figure 7:**
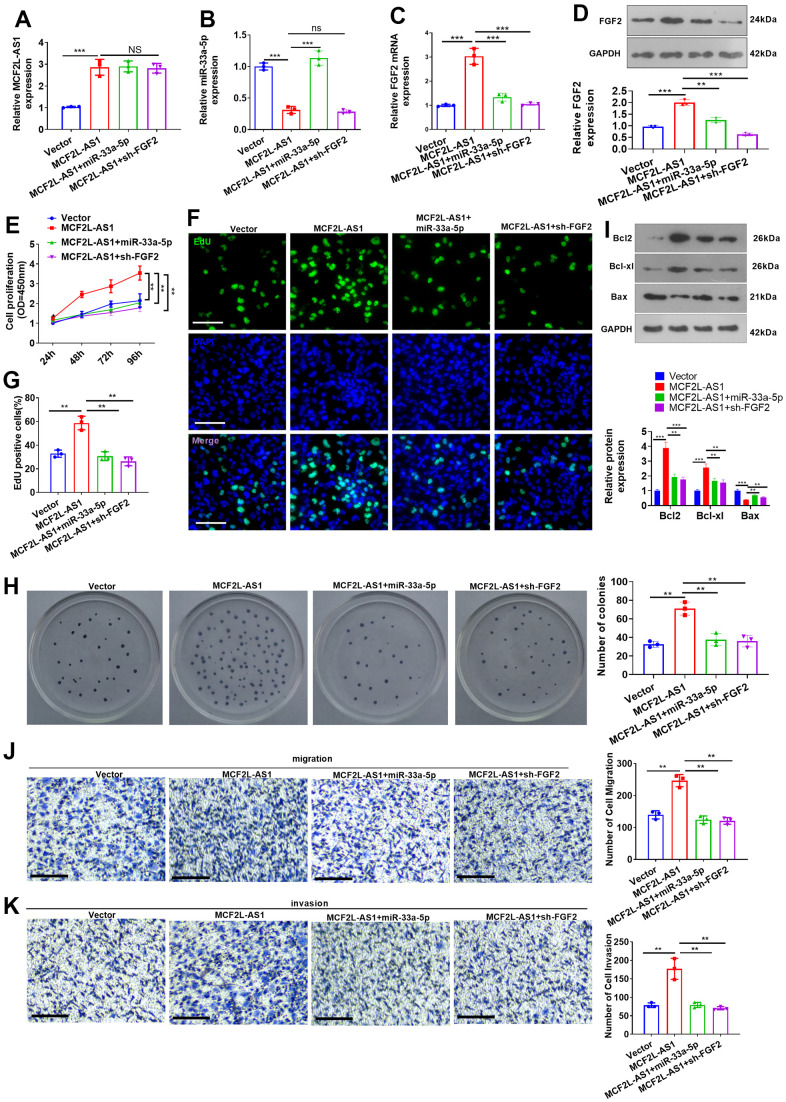
**Effects of MCF2L-AS1-miR-33a-5p-FGF2 on MHCC97H cell proliferation, invasion and apoptosis.** miR-33a-5p mimics or sh-FGF2 or MCF2L-AS1 overexpression plasmids were transfected into MHCC97H cells. (**A**, **B**) qRT-PCR detected MCF2L-AS1and miR-33a-5p expression levels. (**C**) qRT-PCR checked the mRNA level of FGF2. (**D**) Western blot was used for detecting FGF2 expression. (**E**) CCK8 assay detected MHCC97H and HCCLM3 cell proliferation. (**F**, **G**) EdU staining was performed for evaluating cell proliferation. Scale bar=50 μm. (**H**) Colony formation assay was conducted for detecting cell colony formation ability. (**I**) Western blot was conducted for evaluating apoptosis-related proteins, including Bcl2, Bcl-xl, and Bax. (**J**, **K**) Transwell assay detected MHCC97H cell migration and invasion, Scale bar=200 μm. NS P>0.05, * P<0.05, **P<0.01, *** P<0.001. N=3.

**Figure 8:**
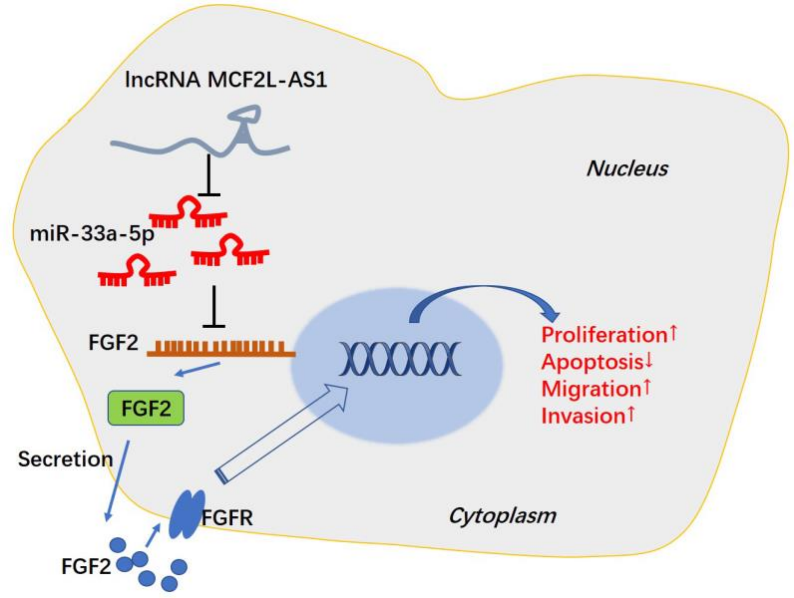
The mechanism of MCF2L-AS1-miR-33a-5p-FGF2 axis in HCC progression.

miR-33a-5p mimics or sh-FGF2 was transfected into MCF2L-AS1-overexpressed MHCC97H cells. We discovered that vis-à-vis the MCF2L-AS1 group, both miR-33a-5p and sh-FGF2 had no significant effect on the MCF2L-AS1 level. miR-33a-5p mimics promoted the miR-33a-5p level and reduced the FGF2 mRNA and protein level (Figure 7A–7D). We monitored cell proliferation *in vitro* through CCK8 assay and unraveled that miR-33a-5p and sh-FGF2 impeded MHCC97H cell proliferation (Figure 7E–7H). We have performed Western blot for evaluating the profiles of apoptosis-related proteins, including Bcl2, Bcl-xl, and Bax. The data showed that MCF2L-AS1 overexpression promoted Bcl2 and Bcl-xl level, while reduced Bax level (Figure 7I). miR-33a-5p mimics or sh-FGF2 addition in MCF2L-AS1-overexpressed cells reduced Bcl2 and Bcl-xl levels and promoted Bax level (Figure 7I). Subsequently, Transwell unveiled that both miR-33a-5p and sh-FGF2 cramped MHCC97H cell invasion and migration (Figure 7J, 7K). All of these findings revealed that MCF2L-AS1-miR-33a-5p-FGF2 might form a regulatory axis in HCC (Figure 8).

## DISCUSSION

Immunotherapy based on targeted molecules has been rapidly developed and shows potential in HCC treatment [27, 28]. Presently, we placed emphasis on the function of MCF2L-AS1 in HCC and found that it derives HCC development via affecting cell proliferation, migration, invasion and apoptosis. The underlying mechanism of MCF2L-AS1 in HCC might depend on regulating the miR-33a-5p-FGF2 axis, which offers a new understanding of HCC progression (Figure 8).

With deeper investigation, the role of MCF2L-AS1 in malignant tumors begins to attract people’s attention [29], and its potential as a tumor diagnostic marker and therapy target is also enhanced [30]. In our study, MCF2L-AS1 profile was abnormally boosted in liver cancer tissues and cells through the database. The survival curve indicated that the high profile of MCF2L-AS1 bore a relation to the poor survival rate of patients. Controlling tumor cell proliferation and metastasis has always been strategies in treating HCC [31, 32]. Therefore, we conducted both *ex vivo* and *in vivo* assays for confirming the function of MCF2L-AS1 in HCC cells. We found that MCF2L-AS1 exerted promotive effects on proliferation, invasion and migration of MHCC97H cells, and reduce apoptosis. These findings denoted that MCF2L-AS1 might be implicated in the pathological process of liver carcinoma.

Competing endogenous RNA (ceRNA) refers to one miRNA that is able to mediate multiple target genes, which constitutes a kind of competitive relationship [33]. RNAs that bind to miRNAs include protein-coding mRNAs, lncRNAs, and some pseudogene transcripts [34, 35]. miR-33a-5p reportedly serves as a tumor-suppressive gene in several tumors, including colon cancer [36] and esophageal cancer [37]. In HCC, miR-33a-5p also has a function. Overexpressing miR-33a-5p increases the cisplatin sensitivity of drug-resistant HCC cells [38]. Another study revealed that aflatoxin B1 enhanced miR-33a-5p, thus inhibiting HCC cell growth and down-regulating β-catenin [39]. Moreover, lncRNAs affect tumor development by targeting and negatively regulating miR-33a-5p [40, 41]. Interestingly, our bioinformatic analysis demonstrated that miR-33a-5p was a potential target of MCF2L-AS1. The decreased expression level of miR-33a-5p was observed in liver cancer tissues, and miR-33a-5p was significantly correlated with the clinical outcomes of patients suffering from HCC. *In vitro* experiments confirmed that miR-33a-5p could contribute to impeded cell proliferation, invasion, and migration. MCF2L-AS1 overexpression led to reduced miR-33a-5p, suggesting a negative regulatory relationship between them.

Fibroblast growth factors (FGFs) are found to be extensively expressed in the tissues of human body; FGF and FGF receptor (FGFR) usually constitute FGFR signaling pathways to participate in growth and development, wound healing and fibrosis, inflammation, and neogenesis of malignant tumors [42]. Altered FGF/FGFR signaling is also observed in HCC [43]. A clinical analysis showed that patients with the FGF2 rs308447 TT genotype had shorter overall survival than patients with the CC or CT genotype (p=0.016) and that FGF2 rs308379 A allele carriers had shorter overall survival than patients with the TT genotype (p=0.020), suggesting that the FGF2 rs308379 A allele and advanced tumor stage were independent prognostic factors for overall survival in patients with HCC [14]. In a recent clinical trial, the potent and selective fibroblast growth factor receptor (FGFR) 1-3 inhibitor pemigatinib has a manageable safety profile and pharmacodynamic and clinical activity in patients with FGFR fusions/rearrangements and mutations [44]. FGF2 has been found to be upregulated in HCC and induces oncogenic functions, such as promoting HCC metastasis, epithelial-mesenchymal transition, and angiogenesis [15, 45, 46]. For example, E2F transcription factor 3 (E2F3) has a combination with the promoter of FGF2 and induces FGF2 overexpression and the malignant progression of HCC cells. The underlying mechanism involves initiating FGFR1-PI3K/AKT and MEK/ERK signaling pathways [47]. Those studies all suggest that FGF2/EGFR pathway is a therapeutic target in HCC. miRNAs have been reported to function in the miRNA-mRNA axis by modulating their own target gene expressions, which modulates biological processes like cell differentiation, proliferation, growth, apoptosis, invasion and migration [41, 48, 49]. Interestingly, our bioinformatic analysis showed that miR-33a-5p can target FGF2 and inhibit its expression at both mRNA and protein levels. Previous studies have revealed that miR-33a-5p can target multiple genes, such as carnitine O-octanoyltransferase (CROT) [50], PTGS2 [40], PNMA family member 1 (PNMA1) [51], and Wnt inhibitors Dickkopf-1 (DKK1) [37] et al. Presently, we confirmed that FGF2 is a direct target of miR-33a-5p, and MCF2L-AS1 enhances FGF2 level by directly targeting miR-33a-5p, and the MCF2L-AS1-miR-33a-5p-FGF2 axis is a potential diagnostic and therapy target in HCC.

However, several limitations also should be noted in our study. First, more clinical samples of HCC are necessary for confirming the predictive role of MCF2L-AS1-miR-33a-5p-FGF2 axis in HCC patients. Second, the underlying mechanism of MCF2L-AS1-miR-33a-5p-FGF2 axis needs investigation. Third, *in vivo* experiments are necessary for confirming this axis in tumor cell growth and metastasis.

## CONCLUSIONS

In summary, we found that MCF2L-AS1 negatively modulated miR-33a-5p and up-regulated FGF2 protein so as to influence MHCC97H proliferation, apoptosis, migration, and invasion. The MCF2L-AS1-miR-33a-5p-FGF2 axis was likely to be implicated in the pathological processes of liver cancer, and MCF2L-AS1 possibly functioned as a potential biomarker for the early screening and diagnosis of cancer.

## MATERIALS AND METHODS

### Clinical sample collection

The tumor specimens and tumor-adjacent normal hepatic tissues were harvested from 53 patients (age: 55.93±12.27) suffering from HCC in HwaMei Hospital, University of Chinese Academy of Sciences. All patients had never received chemotherapy before surgery, and all fresh samples were put into liquid nitrogen after isolation. The patients signed the informed consent. This research was conducted in compliance with the ethical guidelines of the 1975 Declaration of Helsinki and received the green light from the Ethics Committee of HwaMei Hospital, University of Chinese Academy of Sciences (No. 2018124).

### Cell culture

MHCC97H and HCCLM3 (hepatocellular carcinoma cell lines) as well as 293T cells, supplied by Shanghai Cell Bank, Chinese Academy of Sciences, grew in DMEM supplemented with 10 % fetal bovine serum (FBS) (Gibco, Waltham, MA, USA) and 100 U/ml penicillin-streptomycin (Thermo Fisher, Waltham, MA, USA) in a humidified incubator (5% CO_2_, 37° C). The cells in a good state were seeded onto six-well plates (density: 5×10^5^ cells/well). When the cells grew into 60% confluence, they were transfected with MCF2L-AS1 overexpression plasmids, negative controls (miR-NC or vector), miR-33a-5p mimics, or FGF2 knockdown plasmids as per the instructions of transfection reagent Lipofectamine™ 2000 (Life Technologies, Carlsbad, CA, USA).

### Quantitative real-time polymerase chain reaction (qRT-PCR)

TRIzol (Invitrogen, Waltham, MA, USA) was used for extracting total RNA from tissues, MHCC97H and HCCLM3 cells. The total RNA concentration was determined using UV-Vis Spectrophotometry on Thermo Fisher Scientific™ NanoDrop Lite. The integrity of total RNA was confirmed by agarose gel electrophoresis. All extracted RNAs were synthesized into complementary DNA (cDNA) with the utilization of the HiScript III 1st Strand cDNA Synthesis Kit (Vazyme, Nanjing, China). The steps of this procedure were conducted according to the manufacturer’s instructions. Next, RT-qPCR was performed using the SYBR Green PCR kit (Vazyme, Nanjing, China) on a CFX96TM Real-time System (Bio-Rad, Hercules, CA, USA). 45 cycles were included in the reaction, each cycle included amplification at 95° C for 5 minutes, 10 s at 60° C, and 30 s at 72° C. The relative expression of detected genes was analyzed using the 2^-ΔΔCT^ method. GAPDH serves as the internal control of FGF2 and MCF2L-AS1, and U6 was used for the internal control of miR-33a-5p. All experiments were performed in triplicate. The ultimate results were obtained after three repetitions of the tests (Table 2).

**Table 2:** PCR primer sequences.

**Gene**	**Primer sequence**
miR-33a-5p	F:5'-ACACTCCAGCTGGGCAATGTTTCCACAGTG-3'
	R:5'-CTCAACTGGTGTCGTGGAGTCGGCAATTCAGTTGAGGTGATGCA-3'
U6	F:5'-CTCGCTTCGGCAGCACA-3'
	R:5'-AACGCTTCACGAATTTGCGT- 3'
MCF2L-AS1	F:5'-GATCAACGTTCAATCCACCG-3'
	R:5'-ACGTCAAGATAGCGCAGCTTCC -3'
FGF2	F:5'-ATGGCTCCCTTAGCCGAAGT-3'
	R:5'-AGGAAATGCGAACCCACCTG-3'
GAPDH	F:5'-TGTTCGTCATGGGTGTGAAC-3'
	R:5'-ATGGCATGGACTGTGGTCAT-3'

### Dual-luciferase reporter assay

After constructing the wild-type (Wt) and mutant-type (Mut) plasmids of MCF2L-AS1 or FGF2, we extracted MCF2L-AS1-WT/MCF2L-AS1-Mut or FGF2-WT/FGF2-Mut plasmids and transfected them into 293T cells together with miR-33a-5p inhibitors. Subsequent to the removal of the cell culture medium after 48-hour transfection, we washed the cells in PBS and added 20μl lysis buffer for cell lysates collection. The dual-luciferase reporter assay system (Promega, Madison, WI, USA) examined the luciferase activity.

### Cell counting kit-8 (CCK8) assay

MHCC97H and HCCLM3 cells were evenly spread onto 96-well (1×104cells/well) plates with 200 μl of cell suspension per well and were routinely cultured for 24h. As described by the CCK-8 kit’s instructions, 10 μl CCK-8 solution was applied to each well for 1-2h incubation at a temperature of 37° C. A microplate reader (Bio-Rad, Beijing, China) gauged the absorbance (450 nm).

### Colony formation experiment

MHCC97H and HCCLM3 were seeded onto 60 mm dishes with 600 cells per dish. The culture medium was renewed every 2-3 days. Ten days later, the cells were washed by PBS twice and fixed by 4% paraformaldehyde. Crystal violet (0.5%) was used for cell staining. Then the colonies (each colony encompassed over 50 cells) were observed under a light microscope and counted.

### Transwell assay

After the Matrigel gel was kept at 4° C for 12-24 hours, the liquefied Matrigel and the medium were diluted at a ratio of 1:6. The bottom of the upper chamber was coated with Matrigel Diluent (50 μl), which was dried in the incubator for 4h. MHCC97H cells were adjusted to the cell density: 1×106 cells/ml. Then, the upper chamber was added with cell suspension (100 μl). 10% FBS (600 μl) was applied to the medium in the bottom chamber, followed by 24-hour culture in an incubator (5% CO2, 37° C). After removing the chamber, we rinsed it three times with PBS, fixed it with ethanol (95%) for 5 min, and stained it in crystal violet staining solution (0.5%) for 10min. PBS was used to rinse off the staining solution of unbound cells. Cotton swabs were adopted to gently wipe off cells on the upper layer of the filter membrane. The cells on the lower layer were monitored under a microscope (Olympus, Tokyo, Japan).

### EdU assay

The EdU staining kit (Beyotime, Shanghai, China) evaluated cell proliferation. MHCC97H and HCCLM3 cells were grown in 24-well plates (1×105 per well) for 24 hours. The EdU solution was added to each well for 2 hours. PBS flushed the cells twice. After MHCC97H and HCCLM3 were immobilized in 4% paraformaldehyde for 30-60 min and flushed twice in PBS, the cells were incubated along with 0.3% H2O2 (20min, 37° C) and rinsed three times in PBS. Afterwards, MHCC97H cells were incubated for 30 minutes at a temperature of 37° C after the addition of 50μL EdU staining solution. The cells were rinsed 3 times by PBS. The nuclei were stained by DAPI solution (Beyotime, Shanghai, China) and rinsed in PBS (3 times). The EdU-positive cells were mounted for observation under a fluorescence microscope (Olympus, Tokyo, Japan).

### Western blot

Both clinical samples and HCC cells were collected, and the total protein was extracted from the tissues and cells using RIPA lysis (Beyotime, Shanghai, China). After determination of cell concentration, equal content total protein (20 μg) from each group were separated based on molecular weight using gel electrophoresis. Next, the proteins are transferred from the gel onto a PVDF membranes. The membranes were blocked with 5% skim milk to prevent non-specific binding of the primary antibody for one hour at room temperature. Primary antibodies, including Anti-Bcl-2 antibody [E17] (ab32124), Anti-Bcl-XL antibody [E18] (ab32370), Anti-Bax antibody [E63] (ab32503), and Anti-FGF2 antibody [EP1735] (ab92337) were used for incubation at 4° C overnight. The membranes were washed by TBST to remove any unbound primary antibody and then incubated with secondary antibody Goat Anti-Rabbit IgG H&L (HRP) (ab6721) at room temperature for 2 hours. The membranes were washed with TBST to remove any unbound secondary antibody. As for protein detection, the Ultra High Sensitivity ECL Kit (Cat. No.: HY-K1005, MedChemExpress, Monmouth Junction, NJ, USA) was used for bands exposing. The intensity of the band is measured using densitometry to quantify the amount of target protein present.

### Mouse xenograft model

Eighteen BALB/c nude mice (5-9 weeks; 14-21g) were kept at 22° C with 40-75% humidity under a 12h light/dark cycle. Water and food were available for all mice. The nude mice were randomized to two groups: MCF2L-AS1 and negative control (Vector). MHCC97H and HCCLM3 cells were transfected respectively and adjusted to a concentration of 1×107cells/mL. Cell suspension (0.2 ml) was subcutaneously inoculated in the left anterior upper arm armpit of each nude mouse. After inoculation, we measured the max diameter (a) and min diameter (b) of each tumor in the nude mice utilizing a vernier caliper every 3 days in accordance with the formula: V=1/2×a×b2. After 3 weeks, all animals were sacrificed; the tumor was stripped, weighed, photographed and fixed with formaldehyde. The TUNEL staining kit (Beyotime, Shanghai, China) was utilized for evaluating apoptosis in the tumor tissues.

### Analysis of data

Statistical data were exhibited as mean ± standard deviation with the use of the SPSS Statistics 22.0 software. T-test contrasted the statistics of two groups; one-way ANOVA compared data among ≥3 groups. P<0.05 means statistical significance. All tests were duplicated at least 3 times independently.

### Availability of data and materials

The data and materials in the current study are available from the corresponding author on reasonable request.
